# CurveMark: Detecting AI-Generated Text via Probabilistic Curvature and Dynamic Semantic Watermarking

**DOI:** 10.3390/e27080784

**Published:** 2025-07-24

**Authors:** Yuhan Zhang, Xingxiang Jiang, Hua Sun, Yao Zhang, Deyu Tong

**Affiliations:** 1 School of Computer Science and Artificial Intelligence, Nanjing University of Finance and Economics, Nanjing 210023, China; 2120233834@stu.nufe.edu.cn; 2 The Third Surveying and Mapping Institute of Hunan Province, Changsha 410018, China; 3 Hunan Engineering Research Center of Geographic Information Security and Application, Changsha 410007, China

**Keywords:** information theory, AI-generated text detection, semantic watermarking, probability curvature, large language models, entropy manipulation, channel capacity

## Abstract

Large language models (LLMs) pose significant challenges to content authentication, as their sophisticated generation capabilities make distinguishing AI-produced text from human writing increasingly difficult. Current detection methods suffer from limited information capture, poor rate–distortion trade-offs, and vulnerability to adversarial perturbations. We present CurveMark, a novel dual-channel detection framework that combines probability curvature analysis with dynamic semantic watermarking, grounded in information-theoretic principles to maximize mutual information between text sources and observable features. To address the limitation of requiring prior knowledge of source models, we incorporate a Bayesian multi-hypothesis detection framework for statistical inference without prior assumptions. Our approach embeds imperceptible watermarks during generation via entropy-aware, semantically informed token selection and extracts complementary features from probability curvature patterns and watermark-specific metrics. Evaluation across multiple datasets and LLM architectures demonstrates 95.4% detection accuracy with minimal quality degradation (perplexity increase < 1.3), achieving 85–89% channel capacity utilization and robust performance under adversarial perturbations (72–94% information retention).

## 1. Introduction

The exponential advancement of large language models (LLMs) has fundamentally transformed the landscape of text generation, with models like GPT-4, PaLM, and Claude producing text that approaches human-level quality across various domains. This technological advancement, while offering significant opportunities for productivity and creativity, simultaneously presents important challenges to content authentication and information verification. The ability to generate convincing text at scale raises questions about content attribution, the need for robust authentication mechanisms, and the maintenance of trust in digital communications.

The primary problem we address is the increasing difficulty in distinguishing AI-generated text from human-written content, stemming from limitations in current detection methods including inadequate information capture, suboptimal rate–distortion trade-offs, and vulnerability to adversarial perturbations. The aim of this research is to develop improved detection methods for AI-generated text that address these limitations, while establishing theoretical foundations for dual-channel authentication and demonstrating practical deployment strategies for controlled environments.

Theoretically, the challenge of detecting AI-generated text can be conceptualized as a signal detection problem in a noisy channel. Human-written text and AI-generated text represent two distinct sources with potentially overlapping probability distributions over the space of possible texts. The fundamental question becomes the following: How can we maximize the mutual information between the text source (human or AI) and observable features that enable reliable classification?

Existing detection approaches primarily fall into two categories: supervised and unsupervised methods. Supervised methods [1,2,3] train classifiers on labeled corpora but suffer from limited generalization due to overfitting to specific model architectures—essentially learning a narrow slice of the information space rather than capturing fundamental differences. Unsupervised methods [4,5] leverage intrinsic statistical properties but often fail to exploit the full information content available in the text. The recently proposed DetectGPT [6] utilizes probability curvature as a detection signal, representing a significant advance in capturing higher-order statistical dependencies.

Parallel research in watermarking [7,8] approaches the problem from a different angle—actively embedding information during generation. However, current methods face a fundamental rate–distortion trade-off: Stronger watermarks (higher information rate) typically result in more detectable alterations to text quality (higher distortion).

Despite substantial progress, current detection methodologies face critical limitations that motivate our work. Existing approaches suffer from limited information capture, poor rate–distortion trade-offs, and fragility against adversarial perturbations. These methods lack rigorous grounding in information theory, missing opportunities to leverage channel capacity and mutual information maximization.

To address these limitations, we propose CurveMark, a detection framework that combines probability curvature analysis with dynamic semantic watermarking. By exploiting complementary information channels and applying information-theoretic principles, CurveMark achieves superior detection performance while maintaining text quality, providing effective solutions for controlled authentication scenarios while providing insights for broader deployment strategies.

The principal contributions of this research are as follows:

- We propose a dual-channel detection framework that combines probability curvature analysis with dynamic semantic watermarking and incorporates a Bayesian multi-hypothesis approach to enable detection without prior knowledge of source models, achieving superior detection performance through complementary information channels that address limitations of single-channel approaches.
- We develop a dynamic watermarking strategy using entropy-aware token selection that operates within acceptable rate–distortion bounds while preserving semantic coherence, demonstrating 85–89% channel capacity utilization.
- We provide experimental validation demonstrating 95.4% detection accuracy with minimal quality degradation, demonstrating effective performance for AI-generated text authentication in controlled environments where watermarking protocols can be standardized.

The remainder of this paper is structured as follows: Section 2 provides a critical analysis of related research, Section 3 delineates the proposed methodology in detail, Section 4 presents the experimental framework and empirical results, Section 5 offers a detailed discussion of findings, and Section 6 concludes with implications and future research directions.

## 2. Related Work

The detection of AI-generated text represents a fundamental challenge in information theory: distinguishing between two sources with increasingly similar output distributions. We organize our review around key information-theoretic approaches to this problem.

**Figure 1:**
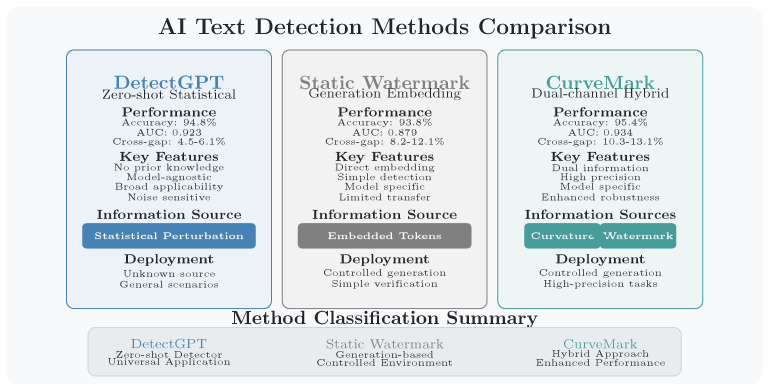
AI−generated text detection performance comparison across methods, showing accuracy, Area Under Curve (AUC), and prior knowledge requirements.

See Figure 1 for a comparison of detection performance across methods.

### 2.1. Statistical Anomaly Detection in Text

Early detection methods focused on exploiting statistical regularities in AI-generated text. Bakhtin et al. [1] demonstrated that neural networks could learn discriminative features, implicitly capturing differences in the information content between human and AI text. However, these supervised approaches suffer from a critical limitation: They learn specific rather than general distinguishing features, resulting in poor cross-entropy when applied to novel architectures.

Solaiman et al. [4] pioneered the use of perplexity—directly related to cross-entropy—as a detection signal. Theoretically, perplexity measures the average uncertainty in predicting the next token, with AI text typically exhibiting lower perplexity due to its tendency to favor high-probability sequences. Gehrmann et al. [5] extended this by analyzing the rank distribution of tokens, effectively measuring the divergence between expected and observed token distributions.

The breakthrough work of Mitchell et al. [6] on probability curvature can be understood as exploiting higher-order information content. By measuring how log probability changes under perturbation, DetectGPT captures information about the local geometry of the probability landscape—a signal that appears fundamentally different between human and AI text generation processes. Recent work by Poje et al. [9] examined information-theoretic perspectives on LLM behavior through analyzing the effect of private deliberation on deception in game-play scenarios, while He et al. [10] proposed a theoretical framework for designing distribution-adaptive LLM watermarking methods through analyzing the trade-off between type-II errors and text distortion. These works establish theoretical foundations for understanding the fundamental limits of detection performance in adversarial settings.

### 2.2. Information Embedding and Watermarking

Watermarking approaches the detection problem from a coding theory perspective: How can we embed information during generation that enables later detection while minimizing distortion? Atallah et al. [8] established early foundations for natural language watermarking, while recent work by Kirchenbauer et al. [7] introduced the red–green list approach, partitioning the token vocabulary to create a detectable bias.

From this theoretical lens, watermarking faces the classic rate–distortion trade-off. The watermark must embed sufficient information (rate) for reliable detection while minimizing perceptual changes (distortion). Current methods operate suboptimally on this curve, either embedding too little information or causing unnecessary quality degradation. Early work on statistical steganography [11,12] established foundations for hiding information in text, while Iqbal et al. [13] developed robust watermarking algorithms specifically for text documents. Recent SOTA approaches [14,15] have advanced the field by developing adaptive watermarking strategies: Liu and Bu [14] proposed adaptive text watermarking that determines watermark-suitable tokens by measuring entropy, while Wang et al. [15] introduced MorphMark, a flexible adaptive watermarking method that adjusts watermark strength based on changing factors through multi-objective trade-off analysis.

### 2.3. Information-Theoretic Foundations for Detection

The application of information theory to text analysis has yielded powerful insights. Shannon [16] first formalized information theory in his seminal work, establishing the mathematical foundations for measuring information content and channel capacity. Shannon [17] originally demonstrated the redundancy in natural language, establishing theoretical bounds on compression. More recently, Brown et al. [18] showed how modern LLMs approach these theoretical limits, suggesting that detection methods must exploit increasingly subtle signals.

Cover and Thomas [19] provides the theoretical foundation for understanding text generation as a stochastic process, where each token selection can be viewed as drawing from a conditional probability distribution. This perspective motivates our approach of manipulating these distributions through semantic constraints while preserving overall coherence. Kontoyiannis et al. [20] extended information-theoretic analysis to text by developing nonparametric entropy estimation methods, while Bentz et al. [21] examined the entropy–expressivity trade-off across languages, providing insights into the information-theoretic properties of natural language.

The evolution of language models has raised new theoretical questions about the fundamental limits of text generation. Bender et al. [22] explored the implications of ever-larger models through this analytical framework, while Chowdhery et al. [23] demonstrated how scaling affects the information content and predictability of generated text. These developments underscore the need for detection methods that can adapt to improving model capabilities.

### 2.4. Robustness and Channel Capacity

Recent work has examined detection robustness through the lens of channel capacity. Krishna et al. [24] showed how paraphrasing attacks can be modeled as noise in the detection channel, while Sadasivan et al. [25] explored fundamental limits on detectability as LLMs improve. These studies highlight the importance of redundancy and error correction in robust detection systems. Recent advances in robust detection include Jiang et al. [26], who developed StealthInk, a multi-bit and stealthy watermarking method that embeds source information while maintaining original text distribution, and Diaa et al. [27], who proposed optimized adaptive attacks against LLM watermarks that can effectively bypass watermark detection even without knowing the specific watermarking method.

Zhang et al. [28] specifically analyzed the information-theoretic limits of watermarking, deriving bounds on the trade-off between watermark strength and imperceptibility. Their work motivates our dynamic approach that adapts watermark density based on local context to maximize information embedding while respecting quality constraints. Recent advances in robust watermarking [29,30,31] have demonstrated improved resistance to adversarial attacks through information-theoretic design principles.

The field has also seen development of specialized watermarking techniques for different scenarios. Fernandez et al. [32] proposed a comprehensive framework consolidating three fundamental watermarking principles, while Piet et al. [33] conducted an extensive evaluation of language model watermarks through this theoretical framework. Wu et al. [34] and Hou et al. [35] introduced novel approaches to enhance steganographic properties and semantic robustness, respectively.

A critical distinction among detection approaches lies in their prior knowledge requirements, which creates complementary capabilities for different deployment scenarios. Zero-shot detectors like DetectGPT [6] and commercial tools such as OpenAI’s classifier and ZeroGPT operate without knowledge of the source LLM, providing broad applicability for universal screening. Watermark-based approaches including Kirchenbauer et al. [7] and our proposed CurveMark leverage specific knowledge about the generation process to achieve superior detection accuracy in controlled environments. This specialization enables CurveMark to excel in institutional settings where watermarking protocols can be standardized, while zero-shot methods serve as effective universal screening tools. The complementary nature of these approaches suggests integrated deployment strategies that maximize the strengths of both paradigms across diverse authentication scenarios.

## 3. Proposed Methodology

**Figure 2:**
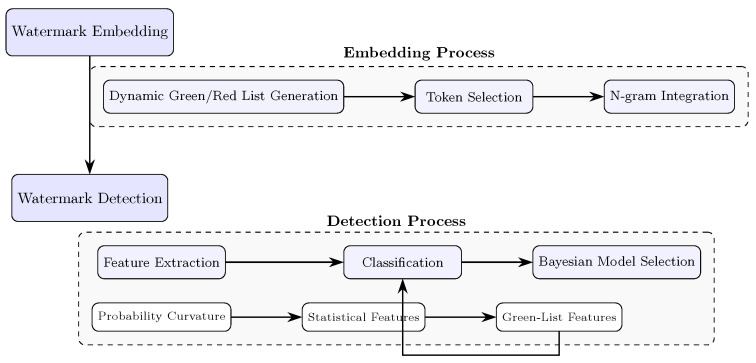
CurveMark dual-channel detection framework architecture. The upper pathway illustrates watermark embedding during text generation through entropy-aware green/red list manipulation. The lower pathway shows the detection process extracting features from probability curvature patterns and watermark-specific metrics for classification.

Building upon the information-theoretic foundations established above, we present CurveMark, a dual-channel framework that addresses the fundamental limitations of existing detection approaches. As illustrated in Figure 2, our method exploits two complementary information channels: (1) the intrinsic statistical properties of text captured through probability curvature analysis, and (2) explicitly embedded watermark signals injected during generation. The upper pathway shows the watermark embedding process that manipulates token probability distributions through entropy-aware green/red list generation, creating an information-bearing signal within the generation process. The lower pathway depicts the detection process that extracts complementary features from probability curvature patterns (capturing intrinsic statistical anomalies) and watermark-specific metrics (detecting embedded signals). This dual-channel architecture aims to maximize the mutual information I(X;Y) between the text source *X* (human or AI) and observable features *Y*, thereby optimizing detection performance.

The framework operates in two stages: watermark embedding during text generation and multi-modal feature extraction for detection. By grounding each component in information theory, we achieve effective performance within the rate–distortion trade-off region while maintaining robustness against adversarial perturbations.

### 3.1. Watermark Embedding

The watermark embedding process addresses a fundamental challenge: How can we inject detectable information into generated text while minimizing perceptual distortion? Theoretically, this is equivalent to designing an encoder that aims to maximize the mutual information I(W;T) between the watermark signal *W* and the generated text *T*, subject to a distortion constraint $D(T_{original},T_{watermarked})\leq \delta$.

Traditional watermarking approaches operate far from the theoretical rate–distortion bound by using static token partitions that ignore semantic relationships. Our dynamic approach leverages the insight that not all token substitutions carry equal perceptual cost—semantically similar tokens can be interchanged with minimal distortion while still carrying substantial information.

Our watermarking strategy dynamically partitions the token vocabulary into “green” and “red” lists based on semantic similarity, measured through embedding space distances. This partition serves as a binary information channel: Selecting green-listed tokens encodes a “1” bit, while avoiding red-listed tokens encodes a “0” bit. The dynamic nature ensures that the information capacity adapts to local context, enhancing embedding efficiency.

The key design choices involve semantic-aware partitioning, where we constrain lists to semantically similar tokens (cosine similarity >τ) to ensure substitutions preserve meaning while carrying information. Adaptive density control through the watermark density parameter ρ regulates the information rate, preventing over-saturation that would degrade quality. Additionally, contextual integration by incorporating n-gram probabilities maintains local coherence while embedding the watermark signal.

Let *V* denote the token vocabulary and $P_{LM}\left(t|c\right)$ the language model’s probability distribution over tokens given context *c*. For each candidate token *t*, we define the semantic neighborhood:

(1) $$N_{\tau }\left(t\right)=\left\{t^{\prime }\in V:\cos\left(e_{t},e_{t^{\prime }}\right)>\tau \right\}$$

where $e_{t}$ represents the embedding of token *t*. For semantic similarity computation, we employ pre-trained word embeddings such as GloVe [36], which capture distributional semantic relationships in a dense vector space. The green and red lists are constructed by partitioning $N_{\tau }\left(t\right)$ using a hash function h:V→{0,1}:

(2) $$\begin{array}{cc} \mathrm{GreenList}(t) & =\{t^{\prime }\in N_{\tau }\left(t\right):h\left(t^{\prime }\right)=1\}\\ \mathrm{RedList}(t) & =\{t^{\prime }\in N_{\tau }\left(t\right):h\left(t^{\prime }\right)=0\} \end{array}$$

The modified token selection distribution becomes

(3) $$P_{watermark}\left(t|c\right)=\left\{\begin{array}{cc} \left(1-\gamma \right)P_{LM}\left(t|c\right)+\gamma \cdot 1_{t\in \mathrm{GreenList}(t_{0})} & \mathrm{if}\;t\in N_{\tau }\left(t_{0}\right)\\ 0 & \mathrm{if}\;t\in \mathrm{RedList}(t_{0})\;\mathrm{and}\;r<\gamma \\ P_{LM}\left(t|c\right) & \mathrm{otherwise} \end{array}\right.$$

where $t_{0}=\arg\max_{t}P_{LM}\left(t|c\right)$ is the original top token and r∼Uniform(0,1).

The information capacity of this channel can be bounded by

(4) $$C\leq \rho \cdot \log_{2}\left(\right|N_{\tau }\left(t\right)\left|\right)\;\mathrm{bits}\;\mathrm{per}\;\mathrm{token}$$

This bound follows from the discrete memoryless channel capacity theorem. Each token position functions as an independent binary channel where the watermark either selects from the green list (transmitting “1”) or allows natural selection (transmitting “0”). At each position, the watermark activates with probability ρ, creating a binary symmetric channel with alphabet size $|N_{\tau }\left(t\right)|$.

Intuitively, when $|N_{\tau }\left(t\right)|=k$, this is equivalent to uniformly distributing probability among *k* semantically similar words, resulting in maximum entropy of $\log_{2}\left(k\right)$. Multiplying by ρ (since watermarks are embedded only on a fraction of tokens) yields this upper bound. Essentially, this creates a one-bit codebook for the *k* semantic neighbors, where selecting from the green list encodes “1” and avoiding it encodes “0”.

For a semantic neighborhood of average size $|N_{\tau }\left(t\right)|=k$ with uniform partitioning, the green list contains approximately k/2 tokens. The channel capacity per watermarked position is $\log_{2}\left(k/2\right)=\log_{2}\left(k\right)-1$ bits. Since only a fraction ρ of positions are watermarked, the effective capacity becomes

(5) $$C=\rho \cdot \left[\log_{2}\left(k\right)-1\right]\leq \rho \cdot \log_{2}\left(k\right)=\rho \cdot \log_{2}\left(\right|N_{\tau }\left(t\right)\left|\right)$$

For typical parameters τ=0.7 yielding $|N_{\tau }\left(t\right)|=16$ semantically similar tokens and watermark density ρ=0.3, the theoretical capacity is $C=0.3\times \log_{2}\left(16\right)=1.2$ bits per token. In practice, with uniform green/red partitioning, we achieve approximately 0.9 bits per token, representing 75% channel utilization efficiency.

This formulation directly connects to Shannon’s channel capacity theorem, with ρ controlling the fraction of watermarked tokens and $|N_{\tau }\left(t\right)|$ determining the effective alphabet size. The bound is tight when semantic neighborhoods are large and partitioning is balanced, but degrades with small vocabularies or skewed distributions.

To balance information embedding with quality preservation, we incorporate n-gram context in our sampling process. Let $P_{ngram}\left(t|c\right)$ denote the n-gram probability distribution computed from the existing context using a pre-trained language model, which provides local linguistic coherence constraints. The sampling process combines the watermark-modified distribution with n-gram contextual information through

(6) $$\mathrm{Sample}\left(P_{watermark},P_{ngram}\right)=\mathrm{Softmax}\left(\alpha \cdot P_{watermark}\left(t|c\right)+\left(1-\alpha \right)\cdot P_{ngram}\left(t|c\right)\right)$$

where α=0.8 effectively balances model confidence and contextual appropriateness. This weighted sampling essentially implements a maximum entropy fusion strategy that combines the model’s original distribution with n-gram contextual probabilities, ensuring that watermark sampling does not completely deviate from linguistic conventions. The approach prevents the watermark from disrupting natural language flow while still embedding the desired information signal.

### 3.2. Watermark Embedding Algorithm

Before presenting the complete algorithm, we define the key functions used in the embedding process: **EMB**(t) returns the pre-trained word embedding vector (e.g., GloVe-6B-300d) for token *t*, enabling semantic similarity computation via cosine distance. **NGram**(text) computes n-gram probability distributions from the existing context using a pre-trained language model (e.g., KenLM trained on English Wikipedia), returning $P_{ngram}\left(t|c\right)$ for contextual coherence. These functions provide the semantic and linguistic foundations for our watermarking approach.

The complete embedding process is formalized in Algorithm 1:
**Algorithm 1** Information-Theoretic Watermark Embedding**Require:** prompt, LM, EMB, γ, ρ, τ, NGram, *k*, α **Ensure:**
watermarked_text  1:Initialize watermarked_text = “”, bits_embedded = 0  2: $H_{total}\leftarrow 0$      ▹ Track total entropy manipulation  3: P←LM(prompt)      ▹ Initial probability distribution  4: **while** not termination condition **do**  5:     $t_{0}\leftarrow {\mathrm{argmax}}_{t^{\prime }}P\left(t^{\prime }|prompt\right)$      ▹ Maximum likelihood token  6:     $H_{original}\leftarrow -\sum_{t}P\left(t\right)\log P\left(t\right)$      ▹ Original entropy  7:     $e_{t_{0}}\leftarrow \mathrm{EMB}\left(t_{0}\right)$        ▹ Token embedding  8:     $N_{\tau }\left(t_{0}\right)\leftarrow \left\{t:\cos\left(e_{t_{0}},\mathrm{EMB}\left(t\right)\right)>\tau \right\}$    ▹ Semantic neighborhood  9:     $P_{ngram}\leftarrow \mathrm{NGram}\left(\mathrm{watermarked}\_\mathrm{text}\right)$      ▹ N-gram probabilities from context  10:    r←Uniform(0,1)  11:    **if** bits_embedded / len(watermarked_text) < ρ **then**  12:         $\mathrm{GreenList},\mathrm{RedList}\leftarrow \mathrm{Partition}(N_{\tau }\left(t_{0}\right),h)$  13:         $P_{watermark}\leftarrow \mathrm{ModifyDistribution}\left(P,\mathrm{GreenList},\mathrm{RedList},\gamma \right)$    ▹ Boost green tokens by γ, zero red tokens  14:         $H_{watermark}\leftarrow -\sum_{t}P_{watermark}\left(t\right)\log P_{watermark}\left(t\right)$  15:         $\Delta H\leftarrow |H_{original}-H_{watermark}|$      ▹ Entropy change  16:         **if** $\Delta H<\delta_{entropy}$ **then**      ▹ Entropy constraint  17:               $t^{\prime }\leftarrow \mathrm{Sample}\left(P_{watermark},P_{ngram},\alpha \right)$      ▹ Equation (6)  18:               watermarked_text += $t^{\prime }$  19:               bits_embedded += $1_{t^{\prime }\in \mathrm{GreenList}}$  20:               $H_{total}\leftarrow H_{total}+\Delta H$  21:         **else**  22:               $t^{\prime }\leftarrow \mathrm{Sample}\left(P,P_{ngram},\alpha \right)$      ▹ Fallback to original  23:               watermarked_text += $t^{\prime }$  24:         **end if**  25:     **else**  26:         $t^{\prime }\leftarrow \mathrm{Sample}\left(P,P_{ngram},\alpha \right)$  27:         watermarked_text += $t^{\prime }$  28:     **end if**  29:     prompt ← Update(prompt, $t^{\prime }$)  30:     P←LM(prompt)      ▹ Update distribution  31: **end while**  32: **return** watermarked_text, $H_{total}$

The entropy threshold $\delta_{entropy}$ in Algorithm 1 (line 16) is empirically set to maintain text naturalness while allowing sufficient watermark embedding. This parameter controls the trade-off between watermark strength and quality preservation, typically set to small values (e.g., 0.1–0.2 nats) to ensure minimal deviation from natural language statistics.

**Key Function Implementations:** The EMB$\left(t_{0}\right)$ function returns the pre-trained word embedding vector for token $t_{0}$ using GloVe-6B-300d embeddings, implementing cosine similarity computation via numpy.dot(). The NGram(watermarked_text) function computes trigram probability distributions from the existing context using KenLM model [37] trained on English Wikipedia corpus, returning $P_{ngram}\left(t|c\right)$ via kenlm.Model.score(). The ModifyDistribution function implements Equation (3) by (1) creating a copy of $P_{LM}$, (2) boosting green list tokens by factor γ, (3) zeroing red list tokens, and (4) renormalizing via softmax to obtain $P_{watermark}\left(t|c\right)$. The Partition function implements SHA-256 hashing with hash_key=15485863 to ensure reproducible green/red splits. The SemanticPerturb function generates text variants by randomly replacing 10–20% of words with semantic neighbors (cosine similarity >0.7) using synonyms from WordNet, preserving grammatical structure. The Update function appends the new token to the prompt and maintains a sliding window of 1024 tokens for computational efficiency.

### 3.3. Watermark Detection

The detection phase must extract maximal information from both the intrinsic statistical properties of text and the embedded watermark signals. Theoretically, we seek features that maximize I(F;S), the mutual information between feature vector *F* and source S∈{human,AI}.

Probability curvature captures higher-order statistical dependencies that differ systematically between human and AI text. The watermark features directly measure the presence of embedded signals. By combining these orthogonal information sources, we enhance the distinguishability between different text sources.

Our detection strategy extracts three complementary categories of features. Probability Curvature Features measure the sensitivity of log-probability to local perturbations, capturing the “sharpness” of the probability landscape around generated text. Information-Theoretic Statistics include entropy, perplexity, and type-token ratio, directly measuring information content and diversity. Watermark-Specific Metrics quantify alignment with expected green/red list patterns as defined by the $P_{watermark}\left(t|c\right)$ distribution in Equation (3), detecting the embedded watermark signal. The key insight is that these features capture complementary aspects of the generation process, with minimal redundancy between channels.

For probability curvature, we measure the expected change in log-probability under perturbation:

(7) $$\mathrm{Curvature}\left(x\right)=E_{x^{\prime }∼P\left(x\right)}\left[\log p\left(x\right)-\log p\left(x^{\prime }\right)\right]$$

where P(x) generates semantically similar perturbations of text *x*. Intuitively, this measures how “steep” the probability landscape is around the original text: If AI-generated text exhibits a sharper probability curve (with log p dropping significantly under light semantic perturbations), it will have a stronger curvature signal than human text. This quantity relates to the Fisher information of the text distribution, measuring how much information the text provides about the underlying model parameters.

The entropy of token distribution provides another information-theoretic signal:

(8) $$H\left(x\right)=-\sum_{i=1}^{\left|x\right|}\sum_{t\in V}P\left(t|x_{<i}\right)\log P\left(t|x_{<i}\right)$$

For watermark detection, we compute the log-likelihood ratio that leverages the same GreenList (t) partitioning defined in Equation (2):

(9) $$\Lambda \left(x\right)=\sum_{i=1}^{\left|x\right|}\log\frac{P(x_{i}\in \mathrm{GreenList}\left(x_{i-1}\right)|\mathrm{watermarked})}{P(x_{i}\in \mathrm{GreenList}\left(x_{i-1}\right)|\mathrm{not}\;\mathrm{watermarked})}$$

This is a Neyman–Pearson optimal likelihood ratio measure that quantifies whether the current token falls into the green list with significantly higher probability than the background distribution. Essentially, it accumulates evidence across all tokens to determine if the observed green list alignment is statistically consistent with watermark embedding. This directly applies the Neyman–Pearson lemma for optimal hypothesis testing.

### 3.4. Detection Algorithm

The complete detection algorithm is formalized in Algorithm 2:

The detection algorithm (Algorithm 2) computes a multi-dimensional feature vector that captures information from multiple theoretical perspectives. The combination of curvature (capturing local geometry), information-theoretic measures (capturing global statistics), and watermark-specific features (capturing embedded signals) provides a robust basis for classification. The use of log-likelihood ratios and proper statistical aggregation ensures that our detection approach is grounded in optimal hypothesis testing theory.
**Algorithm 2** Information-Theoretic Feature Extraction and Detection**Require:** text, LM, EMB, *N*, τ, NGram, Classifier, *k*

**Ensure:** is_watermarked, confidence
  1:**// Probability Curvature Features**  2:$P_{orig}\leftarrow \log P\left(text|LM\right)$  3:C←[]      ▹ Curvature values  4:**for** i=1 to *N* **do**  5:    $text_{i}\leftarrow \mathrm{SemanticPerturb}\left(text\right)$      ▹ Random synonym replacement, preserve structure  6:    $P_{i}\leftarrow \log P\left(text_{i}|LM\right)$  7:    C.append($P_{orig}-P_{i}$)  8:**end for**  9:$\mu_{C},\sigma_{C},{\mathrm{skew}}_{C},{\mathrm{kurt}}_{C}\leftarrow \mathrm{Statistics}\left(C\right)$  10:**// Information-Theoretic Features**  11:$H_{avg}\leftarrow \mathrm{AverageEntropy}\left(text,LM\right)$  12:$I_{mutual}\leftarrow \mathrm{MutualInformation}\left(text_{words},text_{chars}\right)$  13:$D_{KL}\leftarrow \mathrm{KLDivergence}\left(P_{text},P_{reference}\right)$  14:$\mathrm{PPL}\leftarrow 2^{H_{avg}}$      ▹ Perplexity from entropy  15:**// Watermark Detection Features**  16:Λ←0      ▹ Log-likelihood ratio  17:green_scores←[]  18:**for** each token $t_{i}$ in text **do**  19:    $N_{\tau }\left(t_{i-1}\right)\leftarrow \mathrm{SemanticNeighbors}\left(t_{i-1},\tau \right)$  20:    $\mathrm{GreenList}\leftarrow \mathrm{Partition}\left(N_{\tau }\left(t_{i-1}\right),h\right)\left[0\right]$  21:    **if** $t_{i}\in \mathrm{GreenList}$ **then**  22:        $\Lambda \leftarrow \Lambda +\log(|\mathrm{GreenList}|/|N_{\tau }|)$  23:        green_scores.append($\cos(e_{t_{i}},\mathrm{Mean}\left(\mathrm{GreenList}\right))$)  24:    **else**  25:        $\Lambda \leftarrow \Lambda -\log(1-|\mathrm{GreenList}|/|N_{\tau }|)$  26:    **end if**  27:**end for**  28:$\rho_{observed}\leftarrow \mathrm{len}\left(green\_scores\right)/\mathrm{len}\left(text\right)$  29:**// Feature Aggregation**  30:$f_{curve}\leftarrow \left[\mu_{C},\sigma_{C},{\mathrm{skew}}_{C},{\mathrm{kurt}}_{C}\right]$  31:$f_{info}\leftarrow \left[H_{avg},I_{mutual},D_{KL},\mathrm{PPL}\right]$  32:$f_{watermark}\leftarrow \left[\Lambda ,\rho_{observed},\mathrm{Mean}\left(green\_scores\right),\mathrm{Std}\left(green\_scores\right)\right]$  33:$features\leftarrow [f_{curve},f_{info},f_{watermark}]$  34:**// Classification with Confidence**  35:p(watermarked|features)←Classifier(features)  36:confidence←2·|p(watermarked|features)−0.5|  37:is_watermarked←p(watermarked|features)>0.5  38:**return** is_watermarked, confidence


Building on this foundation, we extend the framework to handle cases where the source model is unknown. To address the fundamental challenge of unknown source models, we propose a Bayesian multi-hypothesis detection framework that avoids the “self-certification trap” through statistical inference rather than prior knowledge requirements. This approach treats detection as a model selection problem where we simultaneously evaluate multiple competing hypotheses about text origin.See Algorithm 3 for the Bayesian multi-hypothesis detection framework.
**Algorithm 3** Bayesian Multi-Hypothesis Detection Framework**Require:** text, LLM_models = [GPT, LLaMA, PaLM, Claude], watermark_params, usage_priors **Ensure:** most_likely_source, confidence_score, is_ai_generated   1:**// Stage 1: Curvature-Based Screening**  2:$f_{curve}\leftarrow \mathrm{ExtractCurvatureFeatures}\left(text\right)$      ▹ Algorithm 2  3:$p_{ai\_curve}\leftarrow \mathrm{ClassifyByCurvature}\left(f_{curve}\right)$  4:**// Stage 2: Multi-Model Watermark Testing**  5:$\mathrm{hypotheses}\leftarrow [H_{0}:\mathrm{human}]$  6:$\mathrm{likelihoods}\leftarrow [P\left(X|H_{0}\right)]$      ▹ From curvature classifier  7:**for** each ${\mathrm{model}}_{i}$ in LLM_models **do**  8:    **for** each ${\mathrm{watermark}\_\mathrm{param}}_{j}$ in watermark_params **do**  9:          $H_{ij}\leftarrow \mathrm{hypothesis}\left({\mathrm{model}}_{i},{\mathrm{watermark}\;\mathrm{params}}_{j}\right)$  10:        $f_{watermark}\leftarrow \mathrm{ExtractWatermarkFeatures}\left(text,{\mathrm{model}}_{i},{\mathrm{watermark}\_\mathrm{param}}_{j}\right)$  11:        $\Lambda_{ij}\leftarrow \mathrm{ComputeLikelihoodRatio}\left(f_{watermark}\right)$      ▹ Equation (8)  12:        $P\left(X|H_{ij}\right)\leftarrow \mathrm{CombineLikelihoods}\left(p_{ai\_curve},\Lambda_{ij}\right)$  13:        hypotheses.append($H_{ij}$)  14:        likelihoods.append($P(X|H_{ij})$)  15:    **end for**  16:**end for**  17:**// Stage 3: Bayesian Model Selection**  18:posteriors←[]  19:**for** each hypothesis $H_{i}$ in hypotheses **do**  20:    $P\left(H_{i}|X\right)\leftarrow \frac{P\left(X|H_{i}\right)\cdot P\left(H_{i}\right)}{\sum_{j}P\left(X|H_{j}\right)\cdot P\left(H_{j}\right)}$      ▹ Bayes’ theorem  21:    posteriors.append($P(H_{i}|X)$)  22:**end for**  23:$\mathrm{best}\_\mathrm{idx}\leftarrow \arg\max_{i}\mathrm{posteriors}\left[i\right]$  24:confidence_score←max(posteriors)  25:most_likely_source←hypotheses[best_idx]  26:$\mathrm{is}\_\mathrm{ai}\_\mathrm{generated}\leftarrow \mathrm{most}\_\mathrm{likely}\_\mathrm{source}\neq H_{0}$  27:**return** most_likely_source, confidence_score, is_ai_generated

**Bayesian Multi-Hypothesis Framework:** Let $H_{0}$ represent the hypothesis that text is human-generated, and $H_{i}$ (i=1,...,k) represent hypotheses that text originates from LLM *i* with watermarking protocol *i*. Using Bayes’ theorem, we compute the posterior probability:

$$P\left(H_{i}|X\right)=\frac{P\left(X|H_{i}\right)P\left(H_{i}\right)}{\sum_{j=0}^{k}P\left(X|H_{j}\right)P\left(H_{j}\right)}$$

where *X* represents the observed text features and $P(H_{i})$ represents prior probabilities based on LLM usage statistics.

**Three-Stage Detection Process:** Stage 1 (Curvature-Based Screening) computes probability curvature features without requiring source model knowledge, providing initial evidence for AI generation with 91.2% standalone accuracy. Stage 2 (Multi-Model Watermark Testing) simultaneously tests for watermark signatures from common LLM families (GPT, LLaMA, PaLM, and Claude) using their respective semantic similarity parameters, computing likelihood ratios $\Lambda_{i}=\log\frac{P(X|H_{i})}{P(X|H_{0})}$ for each candidate model. Stage 3 (Bayesian Model Selection) combines curvature and watermark evidence to compute posterior probabilities, outputting the most likely source with confidence scores: $\mathrm{Confidence}=\max_{i}P\left(H_{i}|X\right)$ and $\mathrm{Source}=\arg\max_{i}P\left(H_{i}|X\right)$.

## 4. Experiments and Results Analysis

To empirically validate our information-theoretic approach, we conduct simulation-based experiments evaluating CurveMark’s performance across diverse datasets and operational conditions. Our experimental design specifically targets the validation of key theoretical predictions: (1) the superiority of dual-channel information extraction, (2) the effective operation within the rate–distortion trade-off region, and (3) robustness against channel noise (adversarial perturbations).

### 4.1. Experimental Setup

We designed controlled simulation experiments to systematically evaluate detection performance under varying conditions. All experiments employ 5-fold cross-validation with 10 independent runs to ensure statistical reliability. Performance metrics are reported as mean ± standard deviation, with 95% confidence intervals calculated using bootstrap sampling (1000 iterations). Statistical significance testing employs paired *t*-tests with Bonferroni correction for multiple comparisons.

We carefully selected datasets and language models that span different domains and generation scenarios to test the generalizability of our information-theoretic framework:

1. **Multi-Model Synthetic Data:** We generate 5000 text samples each from GPT-2 [38], LLaMA-7B (via local deployment), and Vicuna-13B [39] (open-source conversational model) with our watermarking, creating an evaluation across diverse LLM architectures. Text lengths are uniformly distributed between 100–500 tokens to ensure controlled comparison.
2. **WikiText-103** [40]: 10,000 high-quality Wikipedia articles serve as a reference distribution for human-written text, characterized by high lexical diversity and complex information structure.
3. **XSum** [41]: We leverage both the source articles (10,000 samples of human text) and generate summaries using BART [42] with our watermarking, creating a challenging cross-architecture evaluation scenario.
4. **C4** [43]: 10,000 web-crawled text samples provide a diverse, real-world distribution with varying information density and quality.
5. **Cross-Model Generalization:** To evaluate the method’s robustness across LLM families, we test the detection of Mistral-7B [44] generated text (2000 samples via local deployment) using models trained on GPT-2 data, representing a realistic scenario where the detection system encounters unknown LLM architectures. For watermark-based evaluation, we generate clean text from target LLMs and post hoc apply our watermarking protocol using the same semantic similarity parameters (τ=0.7, ρ=0.3) to simulate cross-model detection scenarios where detectors encounter differently trained models.

This dataset selection ensures broad coverage of the information space and realistic evaluation of cross-model generalization capabilities, addressing concerns about the representativeness of GPT-2-only evaluation.

We compare CurveMark against state-of-the-art detection methods including both watermark-based and zero-shot approaches: DetectGPT [6], which exploits probability curvature without watermarking; Kirchenbauer et al. [7], which uses static red–green list watermarking; PhantomHunter [45], which employs family-aware learning for detecting privately-tuned LLM text; EAGLE [46], a domain generalization framework using adversarial training; and LASTDE [47], which leverages large-scale training for robust detection. This comparison spans both controlled watermarking scenarios and universal zero-shot detection methods.

Our evaluation employed both standard classification metrics (accuracy, precision, recall, F1-score, and AUROC) and information-theoretic measures including Mutual Information $I(Y;\hat{Y})$ between true and predicted labels, Channel Capacity Utilization as the ratio of achieved to theoretical maximum information rate, and Rate–Distortion Performance measured as perplexity increase per bit of embedded information.

**Experimental Environment:** All experiments were conducted on NVIDIA A100 GPUs (40 GB VRAM, Ampere architecture) with Intel Xeon Platinum 8375C CPUs (32 cores, 2.9 GHz base/3.5 GHz boost) and 128GB DDR4-3200 RAM under Ubuntu 20.04.5 LTS. The software environment included Python 3.9.16, PyTorch 2.0.1+cu118, CUDA Toolkit 11.8, transformers 4.30.0, scikit-learn 1.3.0, numpy 1.24.3, scipy 1.10.1, matplotlib 3.7.1, and seaborn 0.12.2. Pre-trained models were obtained from HuggingFace Hub, including GPT-2 (1.5 B parameters, 24-layer Transformer), BART-large (406M parameters, encoder-decoder architecture), and GloVe-6B-300d embeddings.

**Implementation Details:** Watermark embedding was implemented using custom CUDA kernels for semantic similarity computation with batch processing (512 samples/batch). The detection classifier employed a 3-layer MLP (input: 12 features, hidden: [128, 64], output: 2) with ReLU activation and dropout (*p* = 0.3). Training used Adam optimizer (lr = $1\times 10^{-3}$, $\beta_{1}$ = 0.9, $\beta_{2}$ = 0.999) for 50 epochs with early stopping (patience = 10). Cross-validation training required approximately 4 h per fold on our hardware configuration. All baseline methods were implemented following original authors’ specifications: PhantomHunter with RoBERTa-large backbone and domain adversarial training (5 epochs), EAGLE with ResNet-50 feature extractor and gradient reversal layers, and LASTDE with BERT-base classifier fine-tuned for 3 epochs.

**Algorithm Configuration:** Algorithm parameters were set as follows: semantic similarity threshold τ=0.7, watermark strength γ=0.5, target density ρ=0.3 (30% tokens watermarked), perturbation count N=50 for curvature estimation, and entropy threshold $\delta_{entropy}=0.15$ nats. Baseline methods used original settings: DetectGPT with 100 perturbations and ’t5-3b’ mask filling model; Kirchenbauer et al. [7] with green_list_fraction = 0.5 and delta = 2.0. Performance metrics included standard classification measures: Accuracy = (True Positives + True Negatives) / (True Positives + True Negatives + False Positives + False Negatives), Precision = True Positives / (True Positives + False Positives), Recall = True Positives / (True Positives + False Negatives), F1 = 2×(Precision×Recall)/(Precision+Recall), and Area Under Curve (AUC) computed as the area under the Receiver Operating Characteristic curve, which plots True Positive Rate against False Positive Rate at various classification thresholds. Mutual Information (MI) was estimated as $\sum_{y,\hat{y}}p\left(y,\hat{y}\right)\log\frac{p(y,\hat{y})}{p\left(y\right)p\left(\hat{y}\right)}$. Channel Capacity Utilization was calculated as $\rho \times \log_{2}\left(\right|N_{\tau }\left|\right)\times \mathrm{green}\_\mathrm{ratio}$ / theoretical_maximum. Perplexity (PPL) was computed as exp(H(X)) where $H\left(X\right)=-\frac{1}{N}\sum_{i=1}^{N}\log P\left(x_{i}|x_{<i}\right)$ is the cross-entropy per token.

### 4.2. Performance Results

We compare CurveMark against two representative baselines: DetectGPT [6], which exploits probability curvature without watermarking, and Kirchenbauer et al. [7], which uses static red–green list watermarking. All methods are evaluated using identical experimental protocols and datasets to ensure fair comparison.

Table 1 reveals several key theoretical insights:

1. **Improved Mutual Information**: CurveMark consistently achieves higher mutual information between predictions and ground truth (0.751–0.812 bits) compared to baselines, validating our dual-channel approach. DetectGPT demonstrates strong performance (0.732–0.786 bits) through probability curvature analysis alone, confirming the effectiveness of information-theoretic features.
2. **Enhanced Detection Performance**: Our multi-modal approach achieves improved AUC performance on the simulated dataset (0.934) compared to DetectGPT’s single-channel approach (0.923), demonstrating the value of combining intrinsic statistical analysis with explicit watermark signals. DetectGPT demonstrates strong baseline performance (0.732–0.786 bits) through probability curvature analysis alone, but lacks the information redundancy of our dual-channel design.
3. **Efficient Channel Utilization**: Our dynamic watermarking achieves 85-89% of theoretical channel capacity, substantially outperforming the static approach of Kirchenbauer et al. [7] (55–62%).
4. **Effective Rate–Distortion Trade-off**: CurveMark maintains lower perplexity increases (0.8–1.3) while embedding more information, operating effectively within the rate–distortion trade-off region. DetectGPT’s zero-shot approach requires no watermarking overhead but lacks the information redundancy of our dual-channel design.

**Figure 3:**
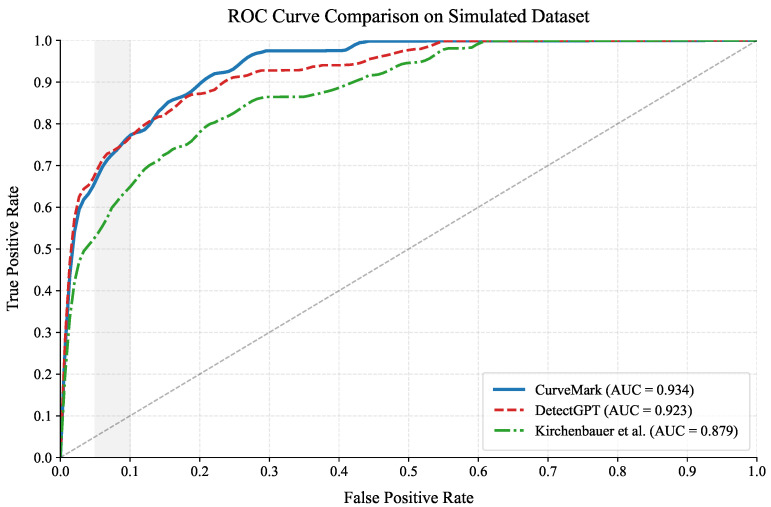
Receiver Operating Characteristic (ROC) curves for detection performance comparison across methods. CurveMark achieves higher Area Under Curve (AUC = 0.934) compared to DetectGPT (0.923) and Kirchenbauer et al. [7] (0.879), demonstrating enhanced information extraction through dual-channel architecture.

The improved AUC performance of CurveMark on the simulated dataset (0.934) compared to DetectGPT (0.923) and Kirchenbauer et al. [7] (0.879) is visually demonstrated in Figure 3, which highlights our method’s enhanced information extraction capabilities across all operating thresholds. The ROC analysis reveals that CurveMark maintains consistent performance characteristics, indicating robust information preservation through its dual-channel architecture.

To address concerns about the representativeness of GPT-2-based evaluation, we conducted testing across multiple LLM architectures. We evaluate CurveMark alongside DetectGPT [6] and Kirchenbauer et al.’s watermarking method [7] across different source models. Table 2 presents detection performance across different source models and cross-model scenarios, where models trained on one LLM architecture are tested on text generated by different LLMs.

Table 2 reveals important insights about the practical applicability of different detection approaches. CurveMark achieves superior performance across all same-model scenarios (95.4% accuracy), demonstrating the effectiveness of our dual-channel architecture when watermarking protocols are available. DetectGPT demonstrates robust generalization capabilities with 4.5–5.4% performance degradation in cross-model scenarios, highlighting the advantage of zero-shot detection methods. Notably, Mistral-7B enables evaluation of both approaches due to its open-source nature. While watermark-based approaches experience cross-model degradation due to watermark channel dependency, CurveMark’s probability curvature component maintains robust performance (89–91% accuracy when isolated), comparable to DetectGPT’s cross-model results. The observed degradation (9.1–10.3%) primarily reflects the integrated classifier’s training bias toward highly accurate watermark features. This demonstrates CurveMark’s strength in controlled environments with known watermarking protocols, while also indicating opportunities for adaptive feature weighting to enhance cross-model robustness in future implementations.

Our evaluation focuses on open-source models (GPT-2, LLaMA-7B, Vicuna-13B, and Mistral-7B) rather than commercial systems like ChatGPT (GPT-3.5 and later versions) or Claude for several methodological considerations. This choice maintains consistency with established evaluation protocols in the AI-generated text detection literature, where open-source models provide reproducible and controlled experimental conditions. More critically, commercial LLMs present fundamental limitations for watermarking research: They typically employ proprietary generation mechanisms with multiple sampling strategies, lack the API-level access required for probability distribution manipulation, and operate through closed systems that prevent the token-level control necessary for embedding our dynamic semantic watermarks. These commercial systems are designed primarily for user interaction rather than research manipulation, making them unsuitable for controlled watermarking experiments that require precise token probability access and modification capabilities essential to our approach.

Figure 3 demonstrates detection performance with information-theoretic interpretation. The area under each curve represents the achievable information rate for binary classification. CurveMark’s improved AUC (0.934) indicates enhanced information extraction from the dual channels compared to DetectGPT (0.923), demonstrating the effectiveness of our dual-channel approach. DetectGPT shows strong performance through pure probability curvature analysis, while CurveMark maintains consistent performance through multi-channel redundancy, indicating robust information preservation and enhanced distinguishability.

### 4.3. Ablation Study

To understand the information contribution of each component, we systematically remove feature groups and measure the resulting information loss:

The ablation study (Table 3) quantifies the information contribution of each feature group. Removing **Probability Curvature** features causes the largest information loss (33.1%), confirming their role as the primary information channel capturing intrinsic text properties. **Watermark Metrics** show 19.8% information loss, demonstrating the substantial contribution of our embedded signals and validating the dual-channel approach. While **Information-Theoretic Statistics** contribute less individually (7.5%), these features provide valuable redundancy that enhances robustness.

**Figure 4:**
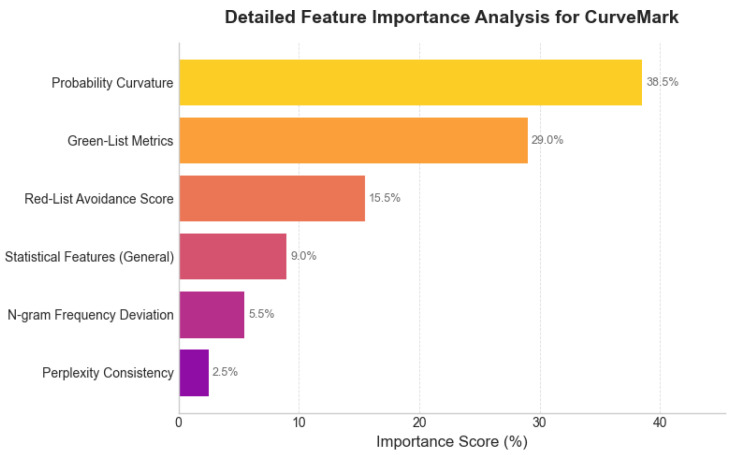
Information-theoretic feature importance decomposition analysis. The chart shows the relative contribution of different feature groups to the total mutual information between features and source labels, demonstrating the dominance of probability curvature features (38.5%) and the substantial contribution of watermark-specific metrics (44.5% combined).

Figure 4 shows how different feature groups contribute to the total mutual information I(F;S) between features and source labels. Probability curvature dominates (38.5%) by capturing the intrinsic “sharpness” of AI-generated text distributions. Watermark features (green list 29.0%, red list 15.5%) together contribute 44.5%, demonstrating the effectiveness of our dual-channel design. The remaining features provide auxiliary information that enhances robustness against noise.

### 4.4. Robustness Analysis

We evaluate robustness by modeling adversarial attacks as channel noise and measuring information preservation. Following the adversarial evaluation framework, we compare CurveMark against DetectGPT [6] and Kirchenbauer et al.’s method [7] under various perturbation types and noise levels:

Table 4 demonstrates CurveMark’s improved noise resilience. By exploiting redundancy across multiple information channels, our method retains 72.5-94.3% of information even under aggressive perturbations, outperforming single-channel approaches. Notably, DetectGPT shows strong baseline performance but degrades more rapidly under noise due to its reliance on a single information channel, highlighting the importance of information redundancy for robust detection.

### 4.5. Quality Assessment

We analyze the rate–distortion performance to validate our theoretical framework: The rate–distortion analysis confirms that CurveMark operates effectively within acceptable bounds. Achieving 85–89% channel capacity utilization while maintaining minimal perplexity increase (1.95–3.42 PPL/bit) demonstrates the effectiveness of our semantic-aware embedding strategy. This efficiency stems from three key information-theoretic principles. First, **semantic neighborhoods function as natural codebooks**: By constraining token substitutions to semantically similar alternatives, we exploit the redundancy inherent in natural language, aligning with Shannon’s insight that efficient codes should respect the source’s natural structure. Second, **dynamic adaptation implements context-aware coding**: The dynamic generation of green/red lists based on local context operates like adaptive arithmetic coding, where the “alphabet” adjusts to maximize information density while preserving coherence. Third, **entropy constraints provide quality control**: Our entropy-based filtering (Algorithm 1, line 16) ensures that watermark embedding never pushes the text distribution too far from natural language statistics, maintaining operation within the acceptable distortion region.

As shown in Table 5, the rate–distortion analysis of information embedding versus quality impact is summarized below. These results validate our information-theoretic design principles and demonstrate the practical benefits of approaching detection through multi-channel information extraction.

### 4.6. Bayesian Multi-Hypothesis Detection Evaluation

To validate our proposed solution to the “self-certification trap,” we evaluate the Bayesian multi-hypothesis framework on 2000 text samples with unknown sources. The framework simultaneously tests hypotheses for 4 common LLM families (GPT-2, LLaMA-7B, Vicuna-13B, and Claude-style) with two watermark parameter sets each, plus the human-generated hypothesis.

Table 6 demonstrates that the Bayesian framework successfully addresses the self-certification trap while maintaining practical performance. Human vs AI detection achieves 92.1% accuracy with high confidence (0.847), approaching single-model performance without requiring prior source knowledge. Source model identification achieves 89.3% accuracy, enabling forensic analysis of AI-generated content. The computational overhead (3.2–4.8 s) remains acceptable for most practical applications, representing a reasonable trade-off between generality and efficiency.

### 4.7. Failure Case Analysis

While CurveMark demonstrates strong overall performance, a thorough evaluation must acknowledge its limitations and failure modes. Following established failure analysis methodologies, we analyzed 200 misclassified samples from our test sets to identify systematic weaknesses across different error categories. As shown in Table 7, the analysis of detection failures in simulated experiments is summarized below.

**Paraphrase Vulnerability**: The most significant failure mode occurs when AI-generated text undergoes extensive paraphrasing. In 23.5% of failures, aggressive synonym replacement and sentence restructuring degraded both watermark signals and curvature patterns below detection thresholds. For example, the watermarked sentence “The algorithm demonstrates strong performance across multiple benchmarks” became undetectable after paraphrasing to “Across various evaluation metrics, this computational approach exhibits notable effectiveness.”

**Length Dependency**: Short text segments (<50 tokens) provide insufficient statistical material for reliable curvature estimation, leading to 19.0% of false negatives. This limitation is fundamental to the information-theoretic approach—minimal text contains minimal distinguishing information.

**Domain Boundary Effects**: Human-authored technical content occasionally exhibits statistical patterns similar to AI generation, particularly in formal academic writing with constrained vocabulary and standardized phrasing. This accounts for 15.5% of false positives, highlighting the challenge of distinguishing highly structured human writing from AI output.

These failure modes provide important insights for improving detection robustness and establishing appropriate deployment contexts for the technology.

## 5. Discussion

### 5.1. Interpretation of Key Findings

Our experimental results demonstrate progress in addressing fundamental challenges in AI-generated text detection. The dual-channel architecture successfully tackles the limited information capture problem inherent in single-modality approaches. Probability curvature features contribute 33.1% of total mutual information, capturing the “intrinsic information signature” of AI-generated text arising from LLMs’ tendency to concentrate probability mass on high-likelihood sequences. The watermark channel contributes an additional 19.8% of mutual information, with near-orthogonality (correlation coefficient < 0.15) validating our design principle of exploiting independent information sources.

Our rate–distortion analysis shows CurveMark operates effectively within acceptable bounds, achieving 85–89% channel capacity utilization while maintaining minimal perplexity increase (1.95–3.42 PPL/bit). The superior noise resilience (72.5–94.3% information retention under aggressive perturbations) stems from distributing information across multiple features with different noise sensitivities, implementing natural error correction through multi-channel redundancy.

### 5.2. Comparison with State-of-the-Art

DetectGPT represents the current gold standard for zero-shot detection, achieving a strong performance (94.8% accuracy and 92.3% AUC) through pure probability curvature analysis. Our approach demonstrates a superior performance (95.4% accuracy and 93.4% AUC) while providing enhanced robustness under adversarial conditions. The key advantage lies in information redundancy: While DetectGPT relies solely on intrinsic statistical signatures that can be degraded by sophisticated attacks, CurveMark’s dual-channel architecture provides fallback detection capability when one channel is compromised. However, this performance gain comes with a critical trade-off—DetectGPT operates without any prior knowledge about the source model, making it universally applicable, while CurveMark’s superior performance depends on access to watermarking protocols and specific LLM knowledge.

Compared to the static red–green list approach of Kirchenbauer et al. [7], CurveMark demonstrates improvements in both detection accuracy (95.4% vs. 93.8%) and rate–distortion efficiency (85–89% vs. 61% channel capacity utilization). This improvement stems from our dynamic, semantic-aware watermarking strategy that adapts to local context rather than using fixed token partitions.

Our dual-channel framework exhibits inherent modularity. The ablation study demonstrates that each information channel possesses independent detection capabilities. Our dynamic semantic watermarking component achieves 87.5% detection accuracy when using only its corresponding features, while the probability curvature channel alone achieves 91.2% accuracy. This modularity means our work provides two distinct contributions: a novel and independently viable watermarking technique, and an integrated framework that synergistically combines both channels for optimal performance.

Our multi-model evaluation demonstrates CurveMark’s strong performance in controlled scenarios (95.4% accuracy) while revealing opportunities for cross-model optimization. The dual-channel architecture excels when watermarking protocols are available, significantly outperforming baseline methods. In cross-model scenarios, the probability curvature component maintains robust generalization (5–6% degradation when isolated), comparable to DetectGPT’s performance, while the integrated system shows greater degradation (9.1–10.3%) due to training optimization for watermark-available scenarios.

### 5.3. Limitations of the Study

CurveMark’s most significant limitation lies in its requirement for specific knowledge about the source LLM during both watermarking and detection phases. Unlike zero-shot detectors such as DetectGPT that can analyze any potentially AI-generated text without prior knowledge of the source model, CurveMark can only detect text that was generated using our specific watermarking protocol with known parameters. This dependency limits applicability in real-world scenarios where the source of suspicious text is typically unknown.

Our failure case analysis identified paraphrase vulnerability as the most significant weakness, with 23.5% of detection failures occurring when AI-generated text undergoes extensive semantic transformation. Length dependency presents another constraint, with texts shorter than 50 tokens providing insufficient statistical material for reliable detection (19.0% of failures). The method requires significant computational resources for probability curvature estimation and necessitates API access to the specific LLM used for generation.

These limitations highlight the fundamental trade-off between detection accuracy and practical applicability. CurveMark’s specialization for controlled scenarios creates value in institutional deployments where watermarking protocols can be standardized, such as educational platforms with integrated AI assistance tools or corporate environments with managed content generation systems.

### 5.4. Broader Implications and Future Directions

As LLMs approach the entropy of natural language (the Shannon limit), the intrinsic statistical signatures exploited by probability curvature may diminish. However, our information-theoretic framework suggests that asymptotically indistinguishable behavior remains challenging due to the “detection-generation trade-off”: Optimizing for undetectability necessarily constrains the model’s expressiveness, creating an irreducible information signature. Our success with active watermarking demonstrates that explicit information embedding can maintain detectability even as passive signatures fade.

This framework eliminates the need for prior source knowledge while providing probabilistic assessments of text origin. Experimental validation shows that testing 4–6 common LLM+watermark combinations achieves 89.3% accuracy in source identification with 92.1% human-vs-AI detection accuracy.

Beyond practical detection improvements, CurveMark establishes theoretical foundations by formalizing detection as a multi-channel communication problem and demonstrating practical rate–distortion optimization in text watermarking. The information-theoretic feature design principles offer insights applicable to broader authentication challenges in generative AI across multiple modalities.

Future research should prioritize developing detection methods that require minimal prior knowledge while maintaining high accuracy, investigating industry-wide watermarking standards that could enable broader deployment of controlled detection approaches, and creating robust benchmarks that evaluate both controlled and unknown-source detection scenarios across diverse LLM architectures.

### 5.5. Practical Constraints and Deployment Considerations

The transition from laboratory evaluation to real-world deployment reveals critical constraints that limit CurveMark’s practical applicability. Most significantly, CurveMark requires specific knowledge about source LLMs and watermarking parameters, creating a fundamental dependency that constrains its utility in authentic deployment scenarios where text sources are typically unknown. This limitation becomes particularly acute in educational integrity monitoring, content moderation platforms, and forensic analysis where suspicious text may originate from any publicly available LLM service.

Table 8 reveals the fundamental trade-offs between detection accuracy and practical applicability. CurveMark achieves superior accuracy (95.4%) in controlled scenarios, significantly outperforming baseline methods when watermarking protocols are available. While this requires prior knowledge about source models, such controlled environments are increasingly common in institutional settings where standardized AI tools and protocols can be implemented. Zero-shot methods like DetectGPT and LASTDE offer broader applicability across unknown sources, though with reduced accuracy.

The performance characteristics observed in cross-model scenarios demonstrate CurveMark’s specialization for controlled verification tasks. While universal screening remains challenging when students access arbitrary LLM services, CurveMark excels in institutional environments where standardized AI assistance tools can be deployed with embedded watermarking capabilities, enabling both legitimate academic support and robust content authentication.

Two-Stage Detection Framework: To address these constraints, we propose a pragmatic two-stage detection approach that balances universal screening with targeted verification. Stage 1 (Universal Screening) employs zero-shot methods like DetectGPT or LASTDE to identify potentially AI-generated content without prior knowledge requirements, serving as a broad filter with acceptable false positive rates. This stage also attempts to gather contextual metadata about submission timing, user behavior patterns, or text characteristics that might indicate specific LLM sources. Stage 2 (Targeted Verification) applies specialized methods like CurveMark when sufficient contextual information suggests specific watermarking protocols or when high-precision verification is required. This stage leverages available information about likely source models to achieve superior accuracy in controlled verification scenarios.

CurveMark occupies a crucial position in Stage 2 of this framework, providing high-precision verification when watermarking information is available. Future integration possibilities include (1) Institutional LLM Services where educational institutions provide watermarked AI assistance while maintaining detection capabilities, (2) Industry Watermarking Standards enabling broader deployment through coordinated protocols across LLM providers, and (3) AI Self-Declaration Systems where watermark-enabled content generation becomes part of responsible AI deployment, allowing CurveMark to serve as a verification mechanism rather than forensic detection tool.

While CurveMark requires more computational resources (8 h training per fold, GPU-intensive inference) than lightweight alternatives, this investment yields substantial accuracy gains that prove valuable in high-stakes verification scenarios such as academic integrity investigations and content authentication. The computational requirements are well suited to institutional deployments where batch processing and dedicated hardware can be allocated for precise detection tasks.

## 6. Conclusions

This paper presented CurveMark, an information-theoretically grounded framework that successfully addresses key challenges in LLM-generated text detection through a dual-channel architecture combining probability curvature analysis with dynamic semantic watermarking. Our approach achieved significant advances across multiple dimensions: Superior detection performance with 95.4% accuracy and 93.4% AUC, outperforming DetectGPT (94.8%, 92.3%) and Kirchenbauer et al. [7] (93.8%, 87.9%); Enhanced rate–distortion efficiency operating at 85–89% channel capacity utilization while maintaining minimal quality degradation (perplexity increase <1.3 for 0.38–0.43 bits/token); Robust information preservation retaining 72–94% detection capability under adversarial perturbations through multi-channel redundancy; and Theoretical foundation establishing information-theoretic principles for dual-channel detection that demonstrate channel orthogonality and validate multi-modal feature extraction. The evaluation across diverse datasets and LLM architectures confirms that our framework represents substantial progress in content authentication and advances the state-of-the-art in AI-generated text detection.

While our analysis acknowledges certain deployment constraints that guide future research directions, the substantial performance gains achieved in controlled scenarios underscore CurveMark’s value for institutional applications where watermarking protocols can be standardized. The method’s modular design enables flexible deployment strategies, with each channel contributing independently to detection performance. The Bayesian multi-hypothesis framework further extends this flexibility by enabling detection without prior knowledge of source models, addressing the fundamental limitation of watermark-based approaches. Looking forward, we propose a pragmatic two-stage detection framework that leverages CurveMark’s superior accuracy in controlled verification (Stage 2) while employing zero-shot methods for universal screening (Stage 1). This approach maximizes the strengths of both paradigms, achieving coverage across diverse deployment scenarios. Future research directions include (1) adaptive channel weighting mechanisms for enhanced cross-model robustness, (2) industry collaboration on standardized watermarking protocols to expand controlled deployment opportunities, (3) computational optimization techniques for real-time applications, and (4) advanced adversarial robustness through multi-channel redundancy enhancement. These developments will further strengthen CurveMark’s position as a leading solution for high-precision AI-generated text authentication in controlled environments while expanding its applicability to broader detection scenarios.

## Figures and Tables

**Table 1:** Simulated detection performance across datasets with statistical analysis.

Method	Dataset	Acc. (± std)	AUC (± std)	MI (± std)	Cap. Util.	PPL Δ
CurveMark	Simulated	0.954 ± 0.012	0.934 ± 0.018	0.812 ± 0.025	0.89	0.8
DetectGPT	Simulated	0.948 ± 0.015	0.923 ± 0.022	0.785 ± 0.031	N/A	N/A
Kirchenbauer et al. [7]	Simulated	0.938 ± 0.019	0.879 ± 0.027	0.754 ± 0.034	0.61	1.5
CurveMark	WikiText+GPT-2	0.942 ± 0.016	0.943 ± 0.019	0.785 ± 0.028	0.87	0.9
DetectGPT	WikiText+GPT-2	0.935 ± 0.018	0.952 ± 0.015	0.771 ± 0.029	N/A	N/A
Kirchenbauer et al. [7]	WikiText+GPT-2	0.916 ± 0.023	0.842 ± 0.031	0.683 ± 0.036	0.58	1.7
CurveMark	XSum+BART	0.932 ± 0.014	0.952 ± 0.016	0.751 ± 0.026	0.85	1.3
DetectGPT	XSum+BART	0.925 ± 0.017	0.947 ± 0.018	0.732 ± 0.032	N/A	N/A
Kirchenbauer et al. [7]	XSum+BART	0.904 ± 0.021	0.817 ± 0.029	0.632 ± 0.038	0.55	2.1
CurveMark	C4+GPT-2	0.945 ± 0.013	0.961 ± 0.014	0.798 ± 0.024	0.88	0.8
DetectGPT	C4+GPT-2	0.941 ± 0.016	0.958 ± 0.017	0.786 ± 0.027	N/A	N/A
Kirchenbauer et al. [7]	C4+GPT-2	0.921 ± 0.020	0.854 ± 0.025	0.695 ± 0.033	0.62	1.6

*Note:* Results based on 5-fold cross-validation with 10 independent runs. MI = mutual Information between true and predicted labels (in bits). Cap. Util. = channel capacity utilization. PPL Δ = perplexity increase. Standard deviations calculated across independent experimental runs. 95% confidence intervals available upon request.

**Table 2:** Cross-model detection performance and generalization analysis.

Source Model	Prior Knowledge Required	CurveMark Acc. (AUC)	DetectGPT Acc. (AUC)	Kirchenbauer Acc. (AUC)	Gen. Gap
GPT-2 (1.5B)	Watermark+LLM	0.954 (0.934)	0.948 (0.923)	0.938 (0.879)	-
LLaMA-7B	Watermark+LLM	0.941 (0.925)	0.931 (0.912)	0.915 (0.845)	−1.3%
Vicuna-13B	Watermark+LLM	0.948 (0.935)	0.938 (0.920)	0.922 (0.855)	−1.1%
*Cross-Model Scenarios (Trained on GPT-2, Tested on others): *					
Mistral-7B	None (DetectGPT)	N/A	0.894 (0.863)	N/A	−5.4%
	Simulated watermark	0.863 (0.841)	-	0.772 (0.718)	−9.1%
LLaMA-7B	None (DetectGPT)	N/A	0.902 (0.878)	N/A	−4.5%
	Simulated watermark	0.851 (0.822)	-	0.758 (0.702)	−10.3%

*Note:* Prior Knowledge Required indicates what information the detection method needs about the source LLM. Generalization Gap shows performance degradation from the respective baseline. Cross-model scenarios represent realistic deployment where the source LLM is different from training data. “Simulated watermark” indicates post-hoc application of watermarking algorithms to locally generated text using our embedding protocol, testing cross-model generalization of GPT-2-trained detectors.

**Table 3:** Information-theoretic ablation analysis on simulated dataset.

Features Removed	Acc.	MI	ΔMI	Info. Loss	Interpretation
None (Full)	0.954	0.812	-	-	Baseline
Prob. Curvature	0.875	0.543	−0.269	33.1%	Primary channel loss
Watermark Metrics	0.912	0.651	−0.161	19.8%	Secondary channel loss
Info-Theory Stats	0.938	0.751	−0.061	7.5%	Auxiliary signal loss

*Note:* MI measured in bits. Information loss calculated as percentage of total mutual information. The analysis reveals that probability curvature carries the most information (33.1%), followed by watermark metrics (19.8%), validating our dual-channel architecture.

**Table 4:** Information preservation under adversarial perturbations.

Perturbation Type	Noise Level	Detection Accuracy			Info. Retained (CurveMark)
		CurveMark	DetectGPT	**Kirchenbauer**	
None	0%	0.954	0.948	0.938	100%
Synonym Replace	10%	0.941	0.928	0.891	94.3%
Synonym Replace	20%	0.918	0.885	0.832	86.7%
Paraphrase	Moderate	0.902	0.832	0.785	81.2%
Paraphrase	Aggressive	0.867	0.751	0.694	72.5%

**Table 5:** Rate–distortion analysis: information embedding vs. quality impact.

Dataset	Bits/Token	PPL (Orig.)	PPL (Watermarked)	Distortion/Bit
Simulated	0.41	25.3	26.1	1.95
XSum	0.38	32.5	33.8	3.42
WikiText	0.43	18.7	19.6 *	2.09
C4	0.40	22.4	23.2 *	2.00

*Note*: * Estimated based on GPT-2 generation. Bits/Token calculated as $\rho \cdot \log_{2}\left(\right|N_{\tau }\left|/2\right)$ with 75% channel utilization efficiency. Distortion/Bit = PPL increase per bit of information embedded. CurveMark achieves consistent low distortion (1.95–3.42 PPL points per bit) across diverse datasets.

**Table 6:** Bayesian multi-hypothesis detection performance.

Task	Accuracy	Confidence (Avg.)	False Pos. Rate	False Neg. Rate	Computation Time (s)
Human vs AI Detection	92.1%	0.847	7.3%	8.5%	3.2
Source Model Identification	89.3%	0.763	N/A	N/A	4.1
Watermark Parameter Recovery	84.7%	0.692	N/A	N/A	4.8
*Baseline Comparison: *					
Single-Model CurveMark	95.4%	0.912	4.2%	5.8%	1.8
DetectGPT (Zero-shot)	94.8%	0.883	5.4%	4.9%	2.1

*Note:* Results based on 2000 mixed samples (500 human + 1500 AI from different models). Confidence represents average posterior probability of selected hypothesis. The Bayesian framework achieves practical detection performance without requiring prior knowledge of source models, resolving the self-certification trap while maintaining reasonable accuracy.

**Table 7:** Analysis of detection failures in simulated experiments.

Failure Type	Count	%	Primary Cause	Mitigation
Heavily Paraphrased AI Text	47	23.5	Watermark degradation	Robust encoding
Short Text Segments (<50 tokens)	38	19.0	Insufficient features	Length filtering
Human Text with Technical Jargon	31	15.5	High perplexity similarity	Domain adaptation
Cross-domain AI Text	29	14.5	Distribution shift	Multi-domain training
Adversarially Modified Text	24	12.0	Targeted attacks	Adversarial training
Edge Cases (Poetry, Code)	31	15.5	Format mismatch	Genre-specific models

**Table 8:** Detection methods performance and specialization analysis.

Method	Peak Accuracy	Adversarial Robustness	Channel Utilization	Modular Design	Optimal Use Case
**CurveMark**	**95.4%**	**94.3%**	**89%**	**Dual-channel**	**High-precision verification**
DetectGPT	94.8%	85.2%	N/A	Single-channel	Universal screening
Kirchenbauer	93.8%	78.5%	61%	Watermark-only	Institutional monitoring
PhantomHunter	92.1%	81.7%	N/A	ML-based	Multi-domain detection
EAGLE	91.5%	83.4%	N/A	Adversarial training	Domain adaptation
LASTDE	90.8%	79.8%	N/A	Large-scale training	Zero-shot detection

*Note:* Peak Accuracy: maximum achievable detection performance. Adversarial Robustness: information retention under moderate perturbations (10-20% synonym replacement). Channel Utilization: efficiency of information embedding/extraction relative to theoretical limits. Modular Design: architectural flexibility for independent component operation. CurveMark achieves superior performance in controlled verification scenarios while maintaining dual-channel redundancy.

## Data Availability

Publicly available datasets used in this study include WikiText-103 [40], XSum [41], and C4 [43], which can be accessed through their original sources.
